# Continuous Flow Techniques in the Total Synthesis of Jaspine B: Part II

**DOI:** 10.1021/acs.joc.5c01799

**Published:** 2025-09-25

**Authors:** Michal Gurský, Dorotea Trnovcová, Pavol Lopatka, Martin Markovič, Peter Koóš, Steven V. Ley, Tibor Gracza

**Affiliations:** † Institute of Organic Chemistry, Catalysis and Petrochemistry, 61791 Slovak University of Technology, Radlinského 9, SK-812 37 Bratislava, Slovak Republic; ‡ Cheminnovatix, Ltd., Račianska 1506/19, SK-831 02 Bratislava, Slovak Republic; § Yusuf Hamied Department of Chemistry, University of Cambridge, Lensfield Road, Cambridge CB2 1EW, U.K.

## Abstract

In this work, we report on the completion of the total synthesis of jaspine B from Garner’s aldehyde using a Pd-catalyzed carbonylation strategy under continuous flow conditions. The target compound was synthesized from the key intermediate bicyclic aminolactone **5** via a sequence of Wittig olefination, DiBAl-H reduction, and hydrogenation, all implemented in flow microreactors. A particularly efficient transfer hydrogenation of the C = C double bond in alkene **8** was achieved by using formic acid as the hydrogen donor and palladium immobilized within a urea-based polymer support (UBPS). Furthermore, a multistep flow system was developed, enabling the direct integration of the hydrogenation of alkene **8** with the final carbamate **9** deprotection step. The methodology was successfully scaled for the preparation of lactol **6** intermediates, demonstrating the potential of continuous flow techniques for the streamlined synthesis of complex natural products.

## Graphical abstract

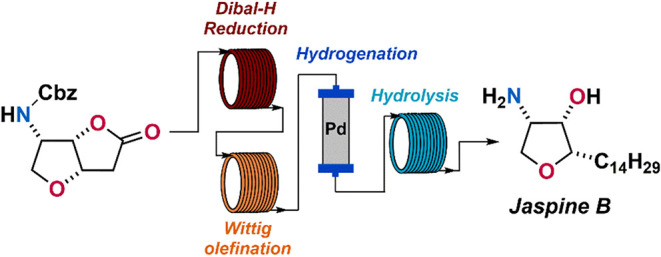

## Introduction

Natural products and secondary metabolites continue to serve as invaluable sources of structurally diverse and biologically active compounds for agrochemical, medicinal, and other industrial applications. Their unique architecture and functionalities have inspired the development of numerous successful therapeutic agents over the past decades.^1^ However, natural products are often isolated in limited quantities, insufficient even for comprehensive biological evaluation, let alone for large-scale studies or clinical development.^2^ Consequently, chemical synthesis remains a crucial strategy to access these compounds in sufficient amounts, supporting both fundamental research and drug discovery efforts.

Despite remarkable advances in synthetic organic chemistry, the preparation of complex natural products remains a formidable challenge. Limitations include lengthy synthetic sequences, low overall yields, and difficulties associated with scalability and reproducibility.^3^ To overcome these obstacles, the development of more efficient, selective, and sustainable synthetic methodologies is essential. In this context, continuous flow chemistry has emerged as a powerful alternative to traditional batch processes.^4–9^ While historically employed in the petrochemical and bulk chemical industries, continuous flow techniques have found widespread application in the synthesis of fine chemicals, natural products, and active pharmaceutical ingredients (APIs),^10,11^ particularly within academic and industrial research settings.^8,9^

Continuous flow systems offer numerous advantages over batch processes, including enhanced heat and mass transfer, precise control over reaction parameters (such as temperature, pressure, and residence time), improved safety, facile scalability, and the possibility of integrating multistep transformations in a seamless, automated manner.^12–16^ These features are particularly advantageous for the synthesis of complex targets where reaction efficiency, selectivity, and reproducibility are critical.

As part of our research program on CO gas-free Pd-catalyzed carbonylation of unsaturated alkenols^17,18^ and their application in natural product synthesis,^19–21^ we previously reported the preparation of jaspine B, a natural sphingolipid of significant biological interest.^22^ The synthesis involved a combination of batch and continuous flow methodologies, starting from commercially available *N*-protected Garner’s aldehyde (Scheme 1).

**Scheme 1:**
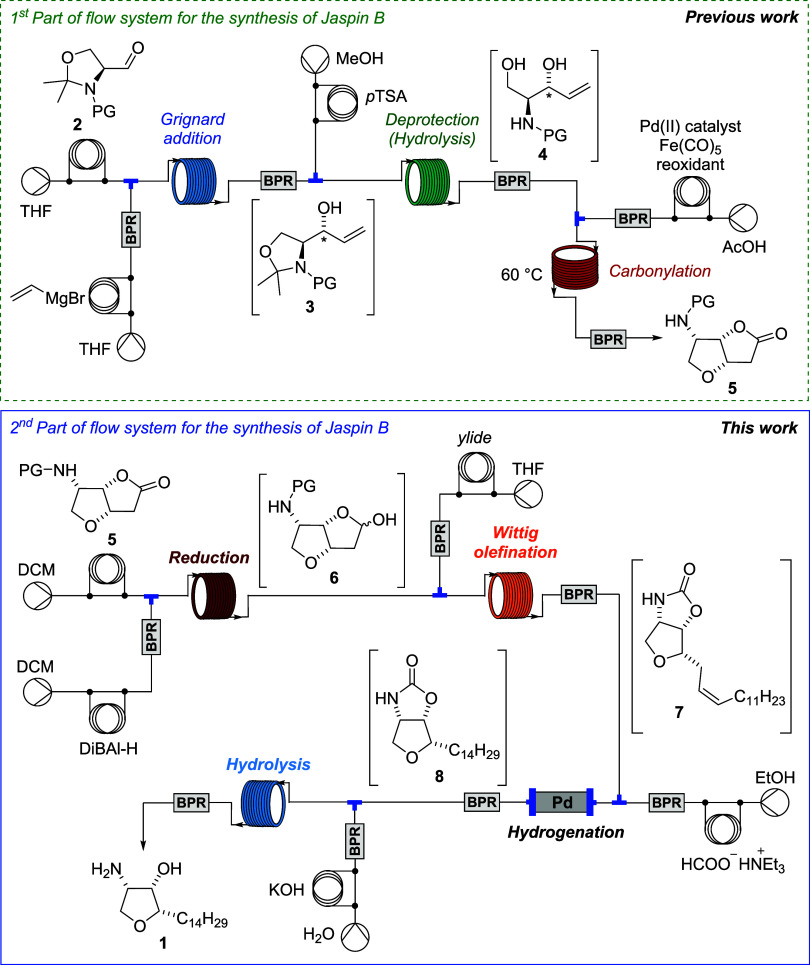
Flow Synthesis of Jaspine B **1**

The key step in the synthesis was the Pd-catalyzed carbonylative cyclization of alcohol **4**, which was carried out in a continuous flow system. The flow reaction at 0.5 mmol scale using a *p*-benzoquinone/LiCl reoxidation system afforded bicyclic lactone **5** in yields comparable to those obtained under batch conditions (Scheme 2).

**Scheme 2:**
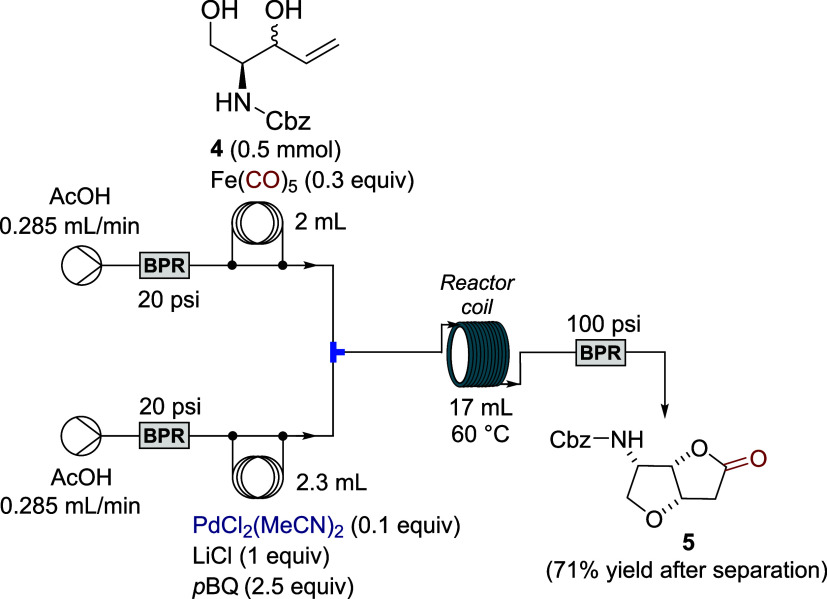
Continuous Pd­(II)-Catalyzed Carbonylation of Unsaturated *N*-Cbz-Protected Aminodiol **4**

To demonstrate the scalability of the method, the large-scale synthesis of *N*-Cbz-**5** was subsequently performed. In this case, the flow setup at a flow rate of 0.785 mL/min of reaction mixture enabled the conversion of 7.6 g of *N*-Cbz-**4** to the corresponding bicyclic lactone in 75% combined yield over a period of 2.5 h.

In this work, we present the development of a second part of a fully integrated continuous flow system for the synthesis of jaspine B, with a focus on the multistep transformation of key intermediates (Scheme 1, this work).

## Results and Discussion

Following the successful large-scale Pd-catalyzed carbonylative cyclization of intermediate *N*-Cbz-**5** under continuous flow conditions,^22^ our attention turned to completing the total synthesis of jaspine B (**1**) by developing a fully continuous flow sequence. The transformation of lactone *N*-Cbz-**5** to the target compound involved a series of key steps: DiBAl-H reduction, Wittig olefination, transfer hydrogenation, and final carbamate deprotection were all adapted for continuous processing (Scheme 1).

Optimization of the flow system commenced with DiBAl-H reduction of lactone *N*-Cbz-**5**. Preliminary batch studies established that a minimum of 1.7 equiv of DiBAl-H was required to achieve complete conversion, while a low temperature of −78 °C was essential to suppress the formation of the undesired over-reduced alcohol *N*-Cbz-**7** (Scheme 3).

**Scheme 3:**
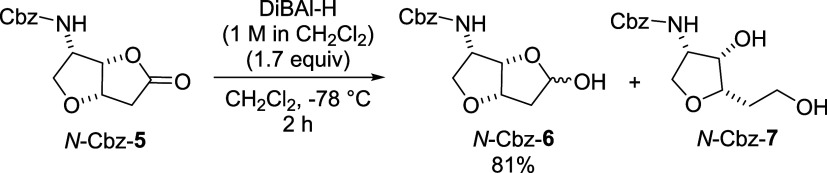
Reduction of Lactone *N*-Cbz-**5**

Based on these findings, a flow setup for the reduction process was proposed (Scheme 4). Thus, solutions of lactones *N*-Cbz-**5** (0.585 M) and DiBAl-H (1.0 M) in anhydrous CH_2_Cl_2_ were introduced into the system via injection coils and pumped at equal flow rates. Both reagent streams were precooled in 2 mL loops prior to combination in a T-mixer, after which the reaction mixture passed through a 10 mL coil reactor. Upon exiting the reactor, the excess of DiBAl-H reagent was quenched by the introduction of methanol. The crude reaction mixture was collected into a saturated aqueous solution of potassium–sodium tartrate to facilitate workup.

**Scheme 4:**
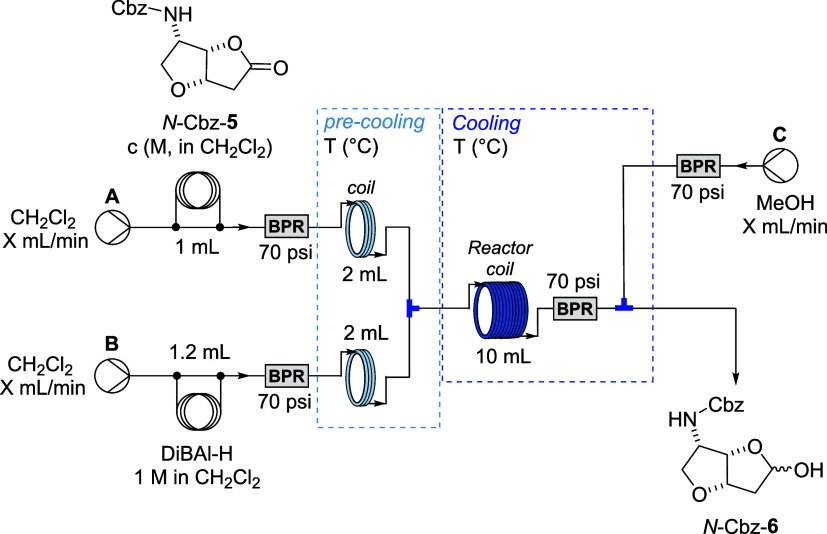
Optimization of the Flow System for the Reduction of *N*-Cbz-**5** Using DiBAl-H Reagent

Published studies indicate that DiBAl-H reductions performed under flow conditions do not require temperatures as low as those typically employed in batch processes.^23,24^ In fact, superior results have been achieved at temperatures between −30 and −45 °C. Moreover, in the initial flow experiments conducted at −78 °C (as applied in the batch conditions), the reaction stream became heterogeneous, resulting in operational challenges in maintaining a stable continuous flow. Based on these observations, we systematically investigated the reduction at various flow rates and temperatures to identify the optimal conditions (Table 1).

**Table 1:** Optimization of *N*-Cbz-5 Reduction in a Flow System^a^

entry	flow rate (mL/min)	DiBAl-H(equiv)	conc. of 5 (M)	reaction time (min)	*T* (°C)	conv.^b^	yield^c^ (%)
1	1	2	0.585	5	–30	full	61
2	0.75	2	0.585	13.3	–40	incomplete	
3	0.5	2	0.585	20	–45	incomplete	58
4^d^	5	4	0.25	1	–45	full	
5^d^	5	3	0.33	1	–45	full	50
6^d^	5	2	0.5	1	–45	full	65
7^d^,^e^	5	2	0.5	1	–45	full	67

a Flow rates of A and B streams were identical.

b Monitored by TLC.

c Isolated yield.

d Flow rate of **C** was 2 mL/min.

e Larger scale: 4.6 mL injection coil for **5**, 5 mL injection coil for DiBAl-H.

The initial flow reduction experiment at −30 °C with a 5 min residence time resulted in full conversion of *N*-Cbz-**5** but afforded only 61% yield of product due to the formation of over-reduced alcohol *N*-Cbz-**7** (Table 1, Entry 1). The formation of alcohol *N*-Cbz-**7** was confirmed by ^1^H NMR of the crude reaction mixture by comparison to known spectral data.^25^ Lowering the reaction temperature to −40 °C and extending the residence time to 13.3 min did not suppress byproduct formation or improve conversion (Entry 2). At −45 °C and 20 min, over-reduction was minimized, though complete conversion was still not achieved (Entry 3). Subsequent experiments at −45 °C with a flow rate of 5 mL/min using 2, 3, or 4 equiv of DiBAl-H resulted in full conversion of the substrate (Entries 4–6). Notably, only the experiment with 2 equiv of DiBAl-H completely suppressed the formation of the over-reduced alcohol **7** (Entry 6).

Finally, the optimized conditions (2 equiv of DiBAl-H at −45 °C, high flow rate) were applied in a larger-scale experiment (Table 1, Entry 7), using injection coils of 4.6 mL for *N*-Cbz-**5** and 5.0 mL for DiBAl-H. Full conversion was achieved within 1 min of the residence time. The crude reaction mixture was collected into a saturated aqueous solution of potassium–sodium tartrate to facilitate workup. However, the isolated yield (67%) was slightly lower than that obtained in the corresponding batch reaction.

With the optimized conditions for the DiBAl-H reduction, we turned our attention to the Wittig olefination. The optimized batch conditions, which involved the use of 4 equiv of ylide and a prolonged reaction time, yielded 81% of the desired carbamate **8** after protection group fragmentation (Scheme 5).

**Scheme 5:**
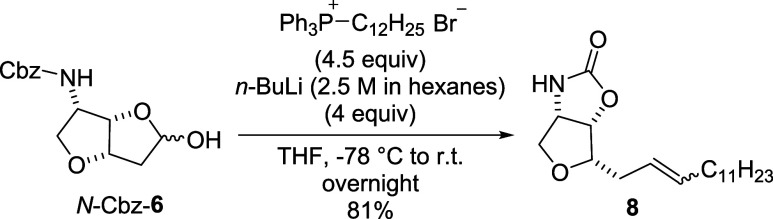
Optimized Wittig Reaction in Batch Arrangement

For the flow system optimization, a basic reaction setup was employed, as illustrated in Scheme 6. While various transformations using organo-phosphorus reagents under flow conditions were reported in the literature, only a few examples of the Wittig olefination are known.^26^ In most cases, stabilized ylides have been generated directly in flow systems by using aqueous solutions of inorganic bases and the corresponding phosphine salts. However, in all reported examples, the limited solubility of both the starting phosphonium salts and the resulting phosphine oxides imposes a significant constraint on these transformations. Newton et al. addressed this limitation by employing a solid-supported phosphonium ylide reagent, thereby circumventing solubility issues and improving the efficiency of the process.^9^ An alternative strategy to overcome these limitations involves the use of a catalytic variant of the Wittig olefination, although this approach has thus far been applied exclusively to stabilized ylides.^27^

**Scheme 6:**
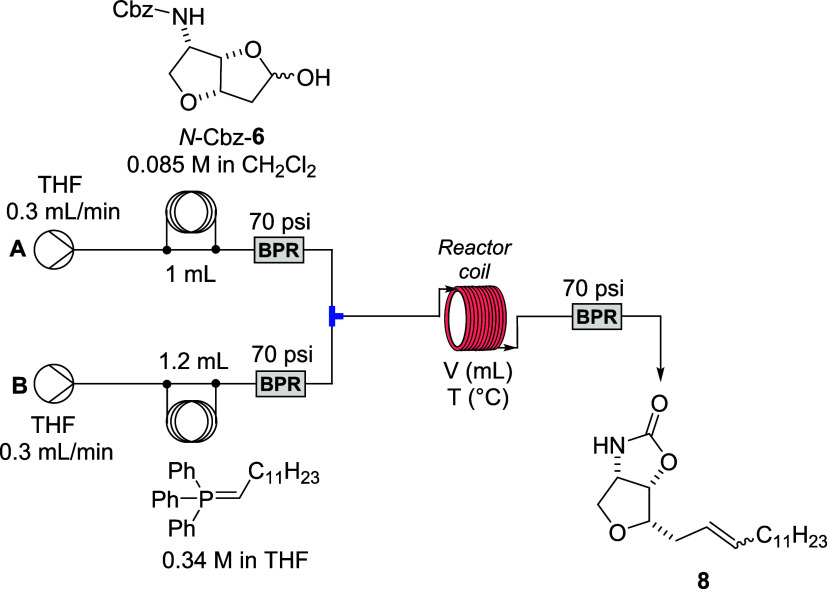
Flow Reactor Used for Optimization of Wittig Reaction

In the case here, the ylide was generated ex situ at −78 °C by the deprotonation of the corresponding phosphonium salt using the *n*-BuLi reagent and used as a solution directly in flow experiments. Solutions of lactol *N*-Cbz-**6** (0.085 M) and the phosphonium ylide (0.34 M) in anhydrous tetrahydrofuran (THF) were introduced into the reactor coil via injection coils (1.0 mL for the substrate and 1.2 mL for the ylide). The relatively high dilution of the ylide was required due to the limited solubility of the phosphonium salt in THF. Both streams were delivered at a flow rate of 0.3 mL/min, combined in a T-mixer, and passed through the reactor coil before exiting the system via a 70 psi back-pressure regulator (BPR).

Initial flow experiment at 70 °C employing a reaction time of 28.3 min did not result in full conversion of lactol *N*-Cbz-**6** (Table 2, Entry 1).

**Table 2:** Reaction Conditions of Wittig Olefination for the Flow System

entry	reaction time (min)	reactor volume (mL)	*T* (°C)	conv.^a^	yield (%)^b^
1	28.3	17	70	incomplete	
2	28.3	17	90	incomplete	
3	50	30	90	full	87
4	50	30	70	full	87

a Monitored by TLC.

b Isolated yield of **8**.

Similarly, the reaction at a higher temperature (90 °C) did not provide full conversion of the substrate (Table 2, Entry 2). Extending the reaction time to 50 min by increasing the reactor volume to 30 mL resulted in full conversion, with the product isolated in an 87% yield (Table 2, Entry 3). Same results were also obtained at a lower reaction temperature (Table 2, Entry 4); however, this experiment was accompanied by pressure buildup in the flow system due to precipitate formation within the reaction coil. In all experiments, the crude reaction mixture was concentrated under reduced pressure, and column chromatography (or filtration through a pad of silica gel) gave the respective product **8**.

Following hydrogenation of the C = C double bond, the reaction was initially performed under batch conditions using hydrogen gas (balloon) and Pd/C, affording the desired product in quantitative yield. However, continuous flow hydrogenation typically requires a mass flow controller or tube-in-tube reactor^28,29^ to safely and accurately introduce gaseous hydrogen,^30^ which was not accessible in our setup. As an alternative, we employed transfer hydrogenation conditions, utilizing a formic acid/triethylamine mixture as the hydrogen donor in combination with various palladium(0) catalysts (Scheme 7 and Table 3).^31^

**Scheme 7:**
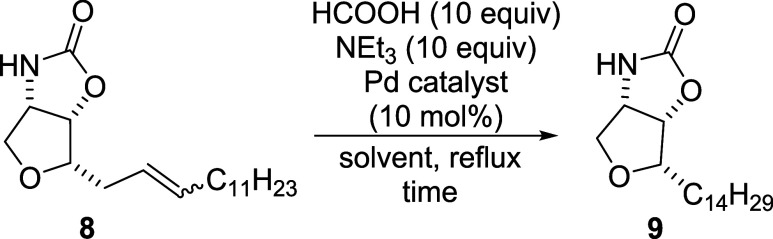
Hydrogenation of Double Bond of **8** Using HCOOH/Et_3_N Hydrogen Donor

**Table 3:** Optimization of Batch Reduction of Intermediate 8 by Transfer Hydrogenation

entry	Pd catalyst	solvent	time (h)	conversion
1	Pd/C	MeOH	1	full
2	Pd EnCat 30NP	MeOH	16	full
3	Pd(0)2@UBPS	MeOH	16	full
4	Pd(0)2@UBPS	EtOH	2	full

Preliminary batch experiments were conducted on a 0.06 mmol scale in sealed vials. Palladium immobilized on carbon (Pd/C) achieved complete conversion within 1 h (Table 3, Entry 1); however, it proved unsuitable for our flow application due to excessive backpressure observed across the catalyst-packed column.^32^

Consequently, alternative heterogeneous catalysts were evaluated,^33^ including palladium encapsulated in a polyurea matrix (Pd EnCat 30NP)^34^ and palladium immobilized on a polymer support (Pd(0)­2@UBPS)^35,36^ (Table 3, Entries 2 and 3). Both systems facilitated full conversion but required extended reaction times. To improve efficiency, the polymer-supported Pd catalyst (Pd(0)­2@UBPS) was tested in a reaction using ethanol, allowing operation at a higher reflux temperature and reducing the batch reaction time to 2 h (Entry 4). Ethanol was also advantageous for downstream compatibility as it is the solvent employed in the subsequent hydrolysis step.

The optimized conditions were then implemented in a continuous flow setup (Scheme 8).

**Scheme 8:**
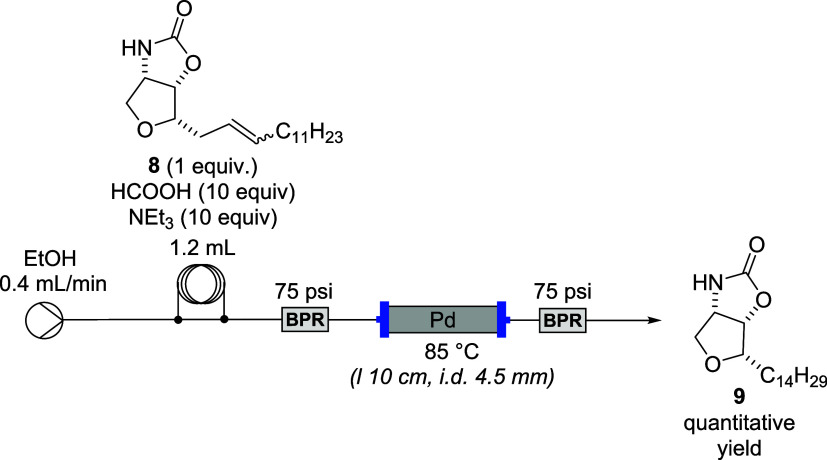
Reduction of Intermediate **8** by Transfer Hydrogenation Under Flow Conditions

A solution of alkene **8**, formic acid, and triethylamine in ethanol was introduced into the system via an injection coil and pumped at a flow rate of 0.4 mL/min. The reaction mixture was passed through a stainless-steel column (10 cm length, 4.5 mm internal diameter) packed with Pd(0)­2UBPS catalyst (305 mg, 2.085 wt %). The output stream was collected over 30 min, and carbamate **7** was isolated by extraction in quantitative yield, as confirmed by NMR analysis.

To streamline the overall transformation, we aimed to integrate the transfer hydrogenation and carbamate hydrolysis into a continuous, telescoped flow sequence. Therefore, a control batch experiment was performed to confirm the compatibility of the optimized carbamate hydrolysis conditions with the preceding transfer hydrogenation step. Thus, a simple batch experiment provided a full conversion of **9** in the hydrolysis step. It was achieved by refluxing the reaction mixture consisting of a 1 M aqueous KOH solution and a filtered hydrogenation reaction mixture. Based on these results, a continuous flow system integrating both steps was constructed (Scheme 9).

**Scheme 9:**
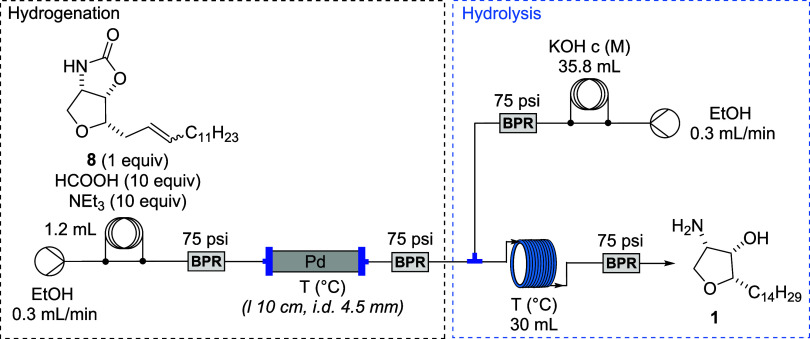
Flow System for Hydrogenation/Hydrolysis Sequence

The hydrogenation part of the flow system remained unchanged from the single-step process, except that the flow rate was reduced to 0.3 mL/min to allow sufficient residence time for the subsequent hydrolysis. After passing through the Upchurch BPR (75 psi), an aqueous KOH stream (Stream B) was introduced via a *T*-junction, and the combined flow was directed into a 30 mL coil reactor. Both the catalyst column and coil reactor were immersed in the same water bath for uniform temperature control. The output stream was collected over a 100 min period.

Initially, the KOH solution was introduced to the system using a syringe pump, but the resulting pressure buildup led to fluctuations in flow rate and inconsistent reagent delivery. To resolve this, the configuration was modified to employ a high-performance liquid chromatography (HPLC) pump for delivering pure ethanol, while the KOH solution was introduced via an injection coil. This modification stabilized the system pressure and ensured a consistent flow and mixing throughout the process.

Initially, a 1 M aqueous KOH solution was introduced into the coil reactor, and the temperature was maintained at the same level as that during the hydrogenation step (Table 4, Entry 1). Under these conditions, full conversion of carbamate **9** was not achieved. Neither increasing the temperature to 95 °C nor increasing the KOH concentration to 2 M resulted in full conversion (Table 4, Entries 2 and 3). Complete conversion was finally achieved by increasing the KOH concentration to 2.5 M, affording jaspine B (**1**) in 65% isolated yield over two continuous steps (Table 4, Entry 4). This yield is comparable to the 67% yield obtained in the corresponding batch sequence.

**Table 4:** Optimization of Sequence Hydrogenation/Hydrolysis

entry	temperature (°C)	KOH aq. solution molality (M)	conversion^a^	yield (%)^b^
1	85	1	incomplete	
2	95	1	incomplete	
3	95	2	incomplete	
4	95	2.5	complete	65

a Detected by TLC.

b Yield of isolated product **8** after two steps.

## Conclusions

In this study, we successfully completed the total synthesis of the bioactive natural product jaspine B (**1**) through a continuous flow approach. The synthetic sequence was initiated from the key intermediate *N*-Cbz-protected bicyclic aminolactone **5**, which had been previously prepared under flow conditions via a Pd-catalyzed carbonylative cyclization. The subsequent transformation of intermediate **5** into Jaspine B was accomplished through four consecutive flow-compatible steps: DiBAl-H reduction, Wittig olefination, transfer hydrogenation, and basic carbamate hydrolysis.

Optimization of the DiBAl-H reduction under flow revealed that complete conversion and selective formation of lactol **6** could be achieved at −45 °C by using 2 equiv of the reducing agent and high flow rates. These conditions were readily scaled using enlarged injection loops, enabling the transformation to be completed in only one minute with a yield of 67%. The Wittig olefination of lactol **6** was successfully translated to flow by using an ex situ-generated ylide. Full conversion to the corresponding alkene **8** was achieved in a 30 mL reactor coil at 90 °C with a residence time of 50 min, yielding the product in 87% isolated yield. The olefination step showed excellent reproducibility and efficiency, making it suitable for scale-up and providing a foundation for further optimization in future studies. Hydrogenation of the C = C bond was efficiently achieved in a packed-bed flow reactor using formic acid/triethylamine as the hydrogen source and Pd^0^ immobilized on a polymeric support (Pd(0)­2@UBPS) as the catalyst. Under optimized conditions (100 °C), alkene **8** was converted quantitatively, with excellent catalyst stability observed throughout the process. The final carbamate deprotection was integrated into the same flow stream using aqueous KOH. Optimization of the second stage revealed that a 2.5 M KOH solution was necessary to achieve complete hydrolysis. A two-stage reactor setup, combining hydrogenation and hydrolysis, allowed the synthesis of Jaspine B in 65% isolated yield over two continuous steps. The flow-based yield was comparable to that obtained under batch conditions (67%).

Overall, this work demonstrates the route of optimization to achieve a successful continuous flow synthesis for the assembly of complex natural products. By integrating multiple transformations into a flow platform, we have improved reaction control, reduced processing times, and achieved yields comparable to or exceeding batch protocols. This study not only completes the total synthesis of jaspine B (**1**) under continuous flow conditions but also highlights general strategies applicable to the scalable preparation of other structurally complex and medicinally relevant molecules.

## Experimental Section

### General Information

Commercial materials, which were obtained from Sigma-Aldrich, Acros Organics, Alfa Aesar, or Fisher Scientific, were used without further purification. Reactions were monitored by using thin-layer chromatography (TLC) on silica gel. Compound purification was undertaken by flash chromatography. All solvents were distilled before use. Hexanes refer to the fraction boiling at 60–65 °C. Flash column liquid chromatography (FLC) was performed on silica gel Kieselgel 60 (15–40 μm, 230–400 mesh), and analytical thin-layer chromatography (TLC) was performed on aluminum plates precoated with either 0.2 mm (DC-Alufolien, Merck) or 0.25 mm silica gel 60 F254 (ALUGRAM SIL G/UV254, Macherey–Nagel). Analyzed compounds were visualized by ultraviolet (UV) fluorescence and by dipping the plates in an aqueous H_2_SO_4_ solution of cerium sulfate/ammonium molybdate, followed by charring with a heat gun. Melting points were obtained using a Boecius apparatus and are uncorrected. ^1^H and ^13^C­{^1^H} NMR spectra were recorded on either 300 (75) MHz MercuryPlus or 600 (151) MHz Unity Inova spectrometers from Varian (Supporting Information). Chemical shifts (δ) are quoted in ppm and are referenced to the tetramethylsilane (TMS), CDCl_3_, or DMSO-d_6_ as an internal standard. Fourier transform infrared (FTIR) spectra were obtained on a Nicolet 5700 spectrometer (Thermo Electron) equipped with a Smart Orbit (diamond crystal ATR) accessory by using the reflectance technique (400–4000 cm^–1^). High-resolution mass spectra (HRMS) were recorded on an Orbitrap Velos mass spectrometer (Thermo Scientific, Waltham, MA; Bremen, Germany) with a heated electrospray ionization (HESI) source. The mass spectrometer was operated with a full scan (50–2000 amu) in positive or negative FT mode (at a resolution of 100,000). The sample was dissolved in methanol and infused via syringe pump at a rate of 5 mL/min. The heated capillary was maintained at 275 °C with a source heater temperature of 50 °C and the sheath, auxiliary, and sweep gases were at 10, 5, and 0 units, respectively. Source voltage was set to 3.5 kV.

### Experimental Procedures

#### Benzyl ((3S,3aS,6aS)-5-Hydroxyhexahydrofuro­[3,2-*b*]­furan-3-yl)­carbamate (N-Cbz-**6**)

The flow setup consisted of three HPLC pumps (Knauer Azura 4.1S with 10 mL pump head) to introduce a solution of lactone (*N*-Cbz-**5**, 0.64 g, 2.3 mmol, 0.5 M) in 4.6 mL of anhydrous CH_2_Cl_2_ (Feed A), a commercial solution of diisobutylaluminum hydride (1.0 M in CH_2_Cl_2_, Sigma-Aldrich, Feed B), and MeOH (Feed C). Injection loops (PTFE, 0.8 mm i.d., 1.6 mm o.d.; internal volume: 4.6 mL, Feed A; and 5 mL, Feed B) were used to deliver the two feeds. Feed C was directly pumped through an HPLC pump. To start the experiment, the complete reactor setup was flushed by pumping anhydrous CH_2_Cl_2_ with flow rates of Feed A = Feed B = 5 mL/min. MeOH was introduced with a Feed C of 2 mL/min. Solutions (Feed A and Feed B) were loaded into their corresponding injection loops and were pumped from the injection loops to precooling loops (PTFE, 0.8 mm i.d., 1.6 mm o.d.; internal volume of Feed A = Feed B = 2 mL) at – 45 °C in an acetone bath. Reaction streams were mixed in a T-shaped connector (PEEK). The combined mixture passed through a coil reactor (PTFE, 0.8 mm i.d., 1.6 mm o.d.; internal volume: 10 mL) at −45 °C and left the reactor through Upchurch BPR (5 bar) before the mixture was combined with Feed C in a T-shaped connector (PEEK) at the same temperature. The final reaction mixture was collected in a flask filled with an appropriate amount of saturated solution of sodium potassium tartrate in water (100 mL) for a period of 7 min. The mixture was left to stir vigorously and subsequently extracted with CH_2_Cl_2_ (3 mL × 40 mL). The residue was purified by MPLC (hexanes/EtOAc: 60/40 to hexanes/EtOAc: 50/50 then isocratic hexanes/EtOAc: 50/50), providing desired lactol *N*-Cbz-**6** (0.41 g, 67%, white solid); *R*_f_ = 0.12 (hexanes/EtOAc, 2:3), mp 96–98 °C. ^1^H NMR (300 MHz, CDCl_3_), major anomer: δ_H_ 7.40 – 7.28 (m, 5H, H_Ar_), 5.69–5.64 (m, 1H, H-3), 5.28 (d, *J* = 7.9 Hz, 1H, NH), 5.11 (s, 2H, PhCH_2_), 4.90 – 4.82 (m, 1H, H-5), 4.69 (t, *J* = 4.8 Hz, 1H, H-1), 4.31 – 4.22 (m, 1H, H-8), 4.04 (t, *J* = 7.7 Hz, 1H, H-7_a_), 3.37 (t, *J* = 8.9 Hz, 1H, H-7_b_), 2.66 (s, 1H, OH), 2.28 – 2.10 (m, 2H, H-4) ppm; minor anomer: δ_H_ 7.40 – 7.28 (m, 5H, H_Ar_), 5.70 (s, 1H, NH), 5.53 (dd, *J* = 8.0, 5.3 Hz, 1H, H-3), 5.11 (s, 2H, PhCH_2_), 4.67 −4.62 (m, 1H, H-5), 4.59 (t, *J* = 4.6 Hz, 1H, H-1), 4.42 – 4.33 (m, 1H, H-8), 3.99–3.88 (m, 2H, H-7), 3.47 (d, *J* = 8.4 Hz, 1H, OH), 2.28 – 2.10 (m, 2H, H-4), ppm. ^13^C­{^1^H} NMR (75 MHz, CDCl_3_), major anomer: δ_C_ 156.3, 136.5, 128.7, 128.6, 128.3, 100.2, 83.2, 82.3, 72.7, 67.1, 53.8, 41.1 ppm; minor anomer: δ_C_ 156.2, 136.4, 128.7, 128.4, 128.3, 100.4, 83.5, 80.6, 69.3, 67.2, 53.8, 42.2 ppm. HRMS (ESI) *m*/*z* [M + Na]+ calcd for C_14_H_17_NNaO_5_ 302.0999, found 302.0994. IR (ATR) ν_max_ 3309, 1691, 1551, 1261, 976 cm^–1^.

#### (3aS,6S,6aS)-6-(Tetradec-2-en-1-yl)­tetrahydrofuro­[3,4-*d*]­oxazol-2­(3H)-one (**8**)

The flow setup consisted of two HPLC pumps (Knauer Azura 4.1S with 10 mL pump head) to introduce a solution of lactol (*N*-Cbz-**6**, 24 mg, 0.085 mmol, 0.085 M) in 1 mL of anhydrous THF (Feed A) and a solution of ylide (0.34 M in THF, Feed B) prepared ex situ according to the procedure described below. Injection loops (PTFE, 0.8 mm i.d., 1.6 mm o.d.; internal volume: 1 mL, Feed A, and 1.2 mL, Feed B) were used to deliver the two feeds. To start the experiment, the complete reactor setup was flushed by pumping anhydrous THF with flow rates of Feed A = Feed B = 5 mL/min. Solutions (Feed A and Feed B) were loaded into their corresponding injection loops and were pumped with flow rates of Feed A = Feed B = 0.3 mL/min and mixed in a T-shaped connector (PEEK). The combined mixture passed through a coil reactor (PTFE, 1.5 mm i.d., 3.2 mm o.d.; internal volume: 30 mL) at 70 °C (reaction coil was immersed in oil bath) and left the system through Upchurch BPR (5 bar). The final reaction mixture was collected in a flask filled with an appropriate amount of saturated solution of NH_4_Cl in water (30 mL) for a period of 70 min. The mixture was extracted with EtOAc (3 mL × 20 mL). The residue was purified by MPLC (hexanes/EtOAc: 100/0 to hexanes/EtOAc: 40/60 then isocratic hexanes/EtOAc: 40:60), providing a mixture of olefins **8** (24 mg, 87%, white solid).

#### Preparation of Ylide Stock Solution

To a solution of dodecyltriphenylphosphonium bromide (1.96 g, 3.8 mmol, 1.3 equiv) in anhydrous THF (8.6 mL) was added dropwise a solution of *n*-BuLi (1.4 mL, 2.5 M in hexanes, 1 equiv) at −78 °C under an Ar atmosphere. The reaction mixture was stirred for 45 min and used afterward without further workup.

#### Characterization of Compound **8**

*R*_f_ = 0.20 (hexanes/EtOAc, 1:4), mp 75–76 °C. ^1^H NMR (300 MHz, CDCl_3_, mixture of *cis/trans* isomers) δ_H_ 5.70 – 5.36 (m, 3H, NH, 6-(H-2 and H-3)), 4.97 (dd, *J* = 7.4, 3.6 Hz, 1H, H-5), 4.38 (dd, *J* = 7.4, 3.7 Hz, 1H, H-1), 3.96 (d, *J* = 10.5 Hz, 1H, H-8_a_), 3.59 – 3.47 (m, 2H, H-8_b_ and H-6), 2.67 – 2.43 (m, 2H, 6-(H-1)), 2.14– 1.95 (m, 2H, 6-(H-4)), 1.47 – 1.14 (m, 18H, 6-(H-5 to H-13)), 0.88 (t, *J* = 6.7 Hz, 3H, 6-(H-14)) ppm.

^13^C­{^1^H} NMR (75 MHz, CDCl_3_, mixture of *cis/trans* isomers) δ_C_ 159.4, 134.6, 133.6, 124.3, 123.7, 83.3, 83.1, 80.9, 73.7, 57.2, 57.1, 32.8, 32.1, 31.6, 29.8, 29.8, 29.7, 29.7, 29.5, 29.5, 29.3, 27.5, 26.5, 22.8, 14.3 ppm. HRMS (ESI) *m*/*z* [M + Na]+ calcd for C_19_H_33_NNaO_3_ 346.2353, found 346.2351. IR (ATR) ν_max_ 2916, 2848, 1757, 1469, 1082 cm^–1^.

#### Jaspine B (**1)**

The flow setup consisted of two HPLC pumps (Knauer Azura 4.1S with 10 mL pump head) to introduce a solution of alkene (**8**, 33 mg, 0.1 mmol, 0.085 M), formic acid (38 μL, 1.0 mmol, 10 equiv) and NEt_3_ (0.14 mL, 1.0 mmol, 10 equiv) in EtOH (Feed A, complete volume of 1.2 mL), and a solution of KOH (2.5 M in water, Feed B). Injection loops (PTFE, 0.8 mm i.d., 1.6 mm o.d.; internal volume: 1.2 mL, Feed A; and PTFE, 1.5 mm i.d., 3.2 mm o.d.; internal volume: 35.8 mL, Feed B) were used to deliver the two feeds. To start the experiment, the complete reactor setup was flushed by pumping EtOH with flow rates of Feed A = Feed B = 300 μL/min. Solutions (Feed A and Feed B) were loaded into their corresponding injection loops. Feed A delivered the solution the injection loop into the stainless-steel column (10 cm length, 4.5 mm internal diameter) filled with Pd immobilized on polymeric support (Pd2(0)@UBPS, 304 mg, 2.085 wt % of Pd) heated to 95 °C (SS column was immersed in oil bath). After Upchurch BPR (75 psi), Feed A was mixed in a T-shaped connector (PEEK) with the KOH solution from Feed B. The combined mixture passed through a coil reactor (PTFE, 1.5 mm i.d., 3.2 mm o.d.; internal volume: 30 mL) at the same temperature (95 °C, coil reactor was immersed in oil bath) before the mixture left the system through Upchurch BPR (75 psi). The final reaction mixture was collected in the flask, and subsequently, the EtOH was evaporated. Water (15 mL) and EtOAc (10 mL) were added to the mixture, the organic phase was separated, and the water phase was extracted with EtOAc (3 mL × 10 mL). Combined organics extracts were dried over anhydrous Na_2_SO_4_, filtered, and concentrated. The residue was purified by MPLC (isocratic CHCl_3_/MeOH/NH_3_ 95:4:1), providing jaspine B (20 mg, 65% over two steps, white solid); *R*_f_ = 0.09 (CHCl_3_/MeOH/NH_3_, 95:4:1), [α]_D_^25^+23.4 (c 1.0, CHCl_3_), mp 92–93 °C. ^1^H NMR (300 MHz, CDCl_3_) δ_H_ 3.92 (dd, *J* = 8.5, 7.3 Hz, 1H, H-5_a_), 3.86 (dd, *J* = 4.9, 3.5 Hz, 1H, H-3), 3.73 (ddd, *J* = 7.2, 6.7, 3.5 Hz, 1H, H-4), 3.70 – 3.61 (m, 1H, H-2), 3.51 (dd, *J* = 8.5, 6.7 Hz, 1H, H-5_b_), 1.41 (s, 1H, OH), 1.38 – 1.19 (m, 24H, 2-(H-2 to H-13)), 1.11 (s, 2H, NH_2_), 0.88 (t, *J* = 6.7 Hz, 3H, 2-(H-14)) ppm. ^13^C­{^1^H} NMR (75 MHz, CDCl_3_) δ_C_ 83.4, 72.5, 71.9, 54.4, 32.1, 30.0, 29.8, 29.8, 29.8, 29.7, 29.6, 29.5, 26.5, 22.9, 14.3 ppm. HRMS (ESI) *m*/*z* [M + H]+ calcd for C_18_H_38_NO_2_ 300.2897, found 300.2893. IR (ATR) ν_max_ 2916, 2849, 1722, 1467, 873 cm^–1^.

## Supplementary Material



## Data Availability

The data underlying this study are available in the published article and its Supporting Information.

## Notes

Copies of ^1^H and ^13^C­{^1^H} NMR spectra for all prepared compounds (PDF)
