# Supplementary material for: Continuous Flow Techniques in the Total Synthesis of Jaspine B: Part II
Source: J Org Chem. 2025 Sep 25;90(40):14186–94. doi: 10.1021/acs.joc.5c01799 (PMC12519474; doi:10.1021/acs.joc.5c01799)
Supplement: Supplementary file 1 [file jo5c01799_si_001.pdf]

Continuous Flow Techniques in the Total Synthesis of Jaspine B: Part II

Michal Gurský,[1] Dorotea Trnovcová,[1] Pavol Lopatka,[1] Martin Markovič,[1,2] Peter Koóš,[1,2,*] Steven V. Ley[3] and Tibor Gracza[1]

[1] Institute of Organic Chemistry, Catalysis and Petrochemistry, Slovak University of Technology, Radlinského 9, SK-812 37 Bratislava, Slovak Republic.

[2] Cheminnovatix, Ltd., Račianska 1506/19, SK-831 02 Bratislava, Slovak Republic; info@cheminnovatix.com

[3] Yusuf Hamied Department of Chemistry, University of Cambridge, Lensfield Road, Cambridge CB2 1EW, U.K.

* Corresponding authors: peter.koos@stuba.sk (P.K.); Tel.: +421 2 5932 5129 (P.K.)

Supporting information

Table of contents

Copies of ^{1}H and ^{13}C NMR spectra S2

Copies of ^{1}H and ^{13}C NMR spectra

^{1}H, $^{13}C\{^{1}H\}$ NMR spectrum of compound *N*-Cbz-**6** ……………………………..……….S3-S4

^{1}H, $^{13}C\{^{1}H\}$ NMR spectrum of compound **8** ……………………………..………………S5-S6

^{1}H, $^{13}C\{^{1}H\}$ NMR spectrum of compound **1** ……………………………………………...S7-S8

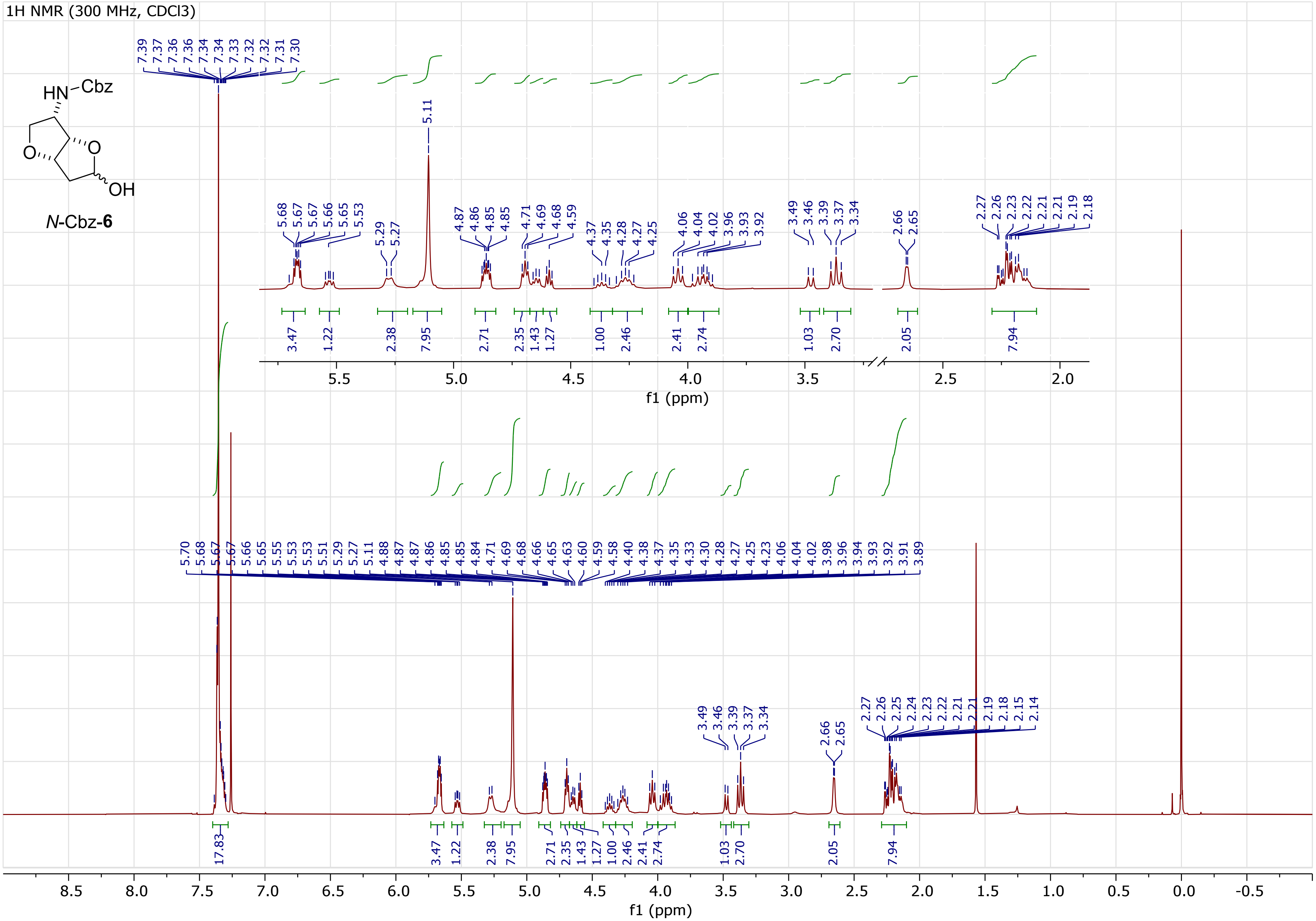
1H NMR (300 MHz, CDCl3)
HN
Cbz
O
O
OH
N-Cbz-6
f1 (ppm)
f1 (ppm)

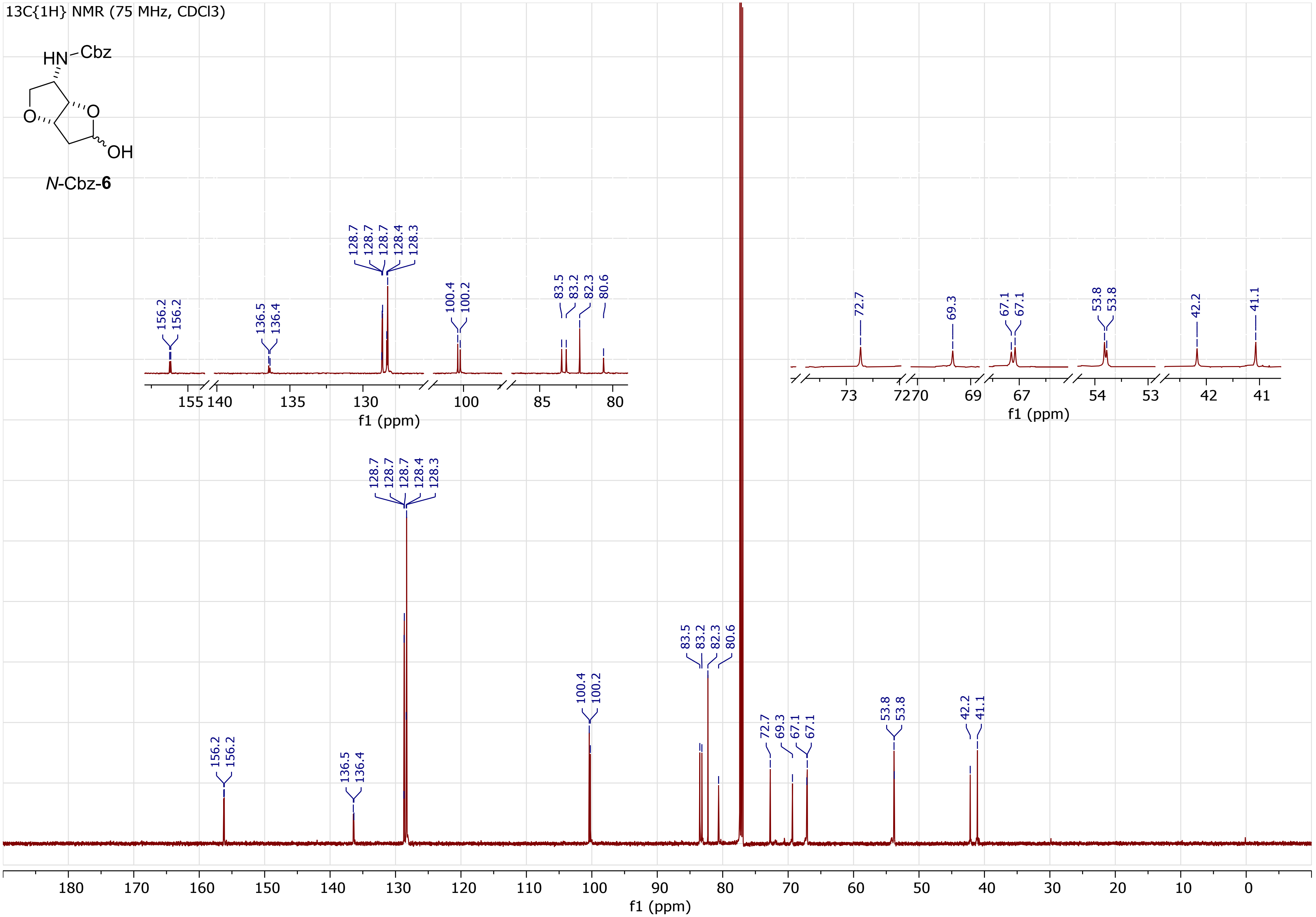
13C{1H} NMR (75 MHz, CDCl3)
HN–Cbz
O
O
OH
N-Cbz-6
156.2
156.2
136.5
136.4
128.7
128.7
128.7
128.4
128.3
100.4
100.2
83.5
83.2
82.3
80.6
72.7
69.3
67.1
67.1
53.8
53.8
42.2
41.1
f1 (ppm)

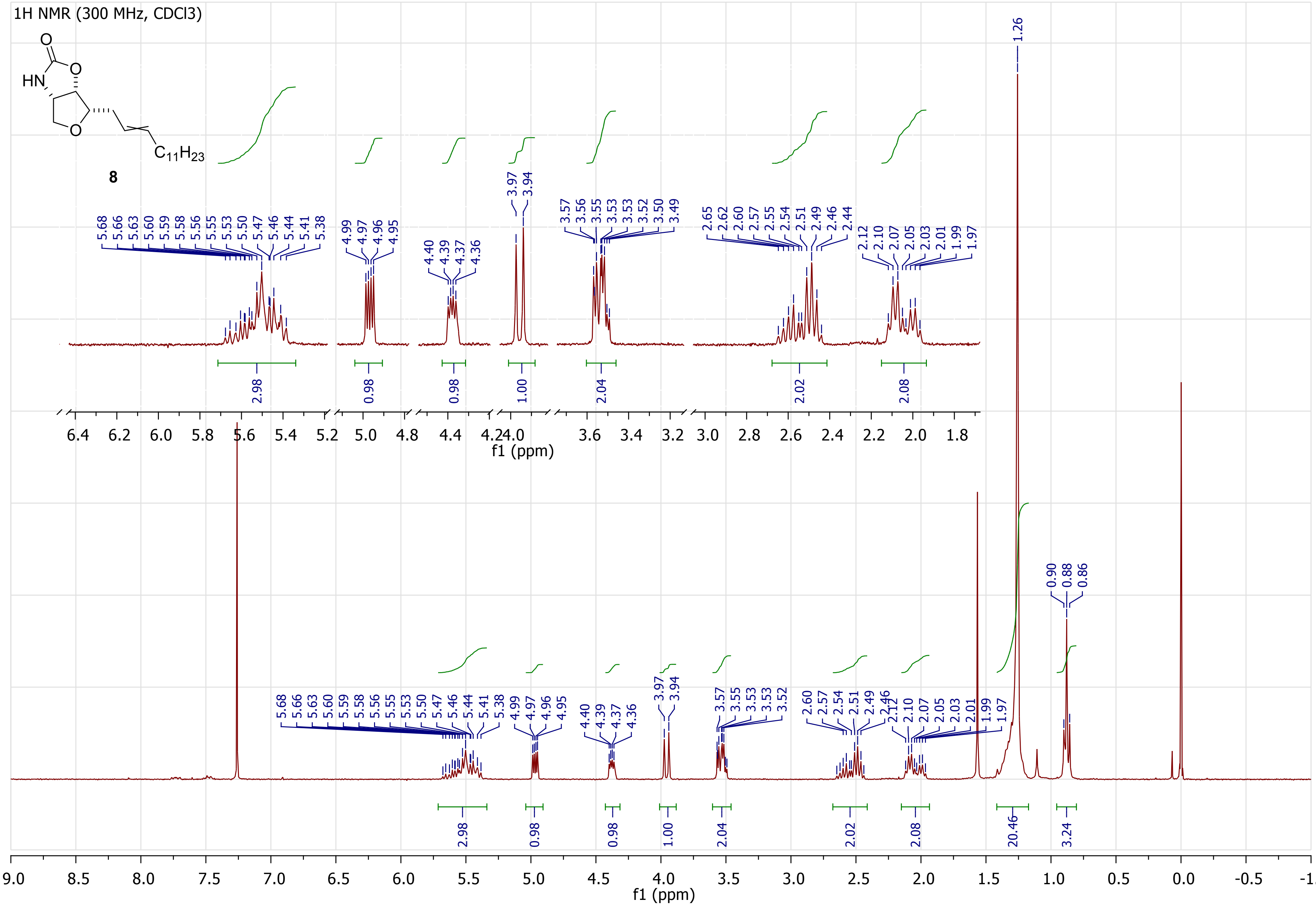
1H NMR (300 MHz, CDCl3)
HN
O
O
O
C11H23
8
f1 (ppm)
2.98
0.98
0.98
1.00
2.04
2.02
2.08
20.46
3.24

13C{1H} NMR (75 MHz, CDCl3)

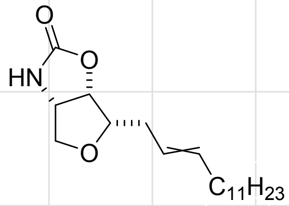

8

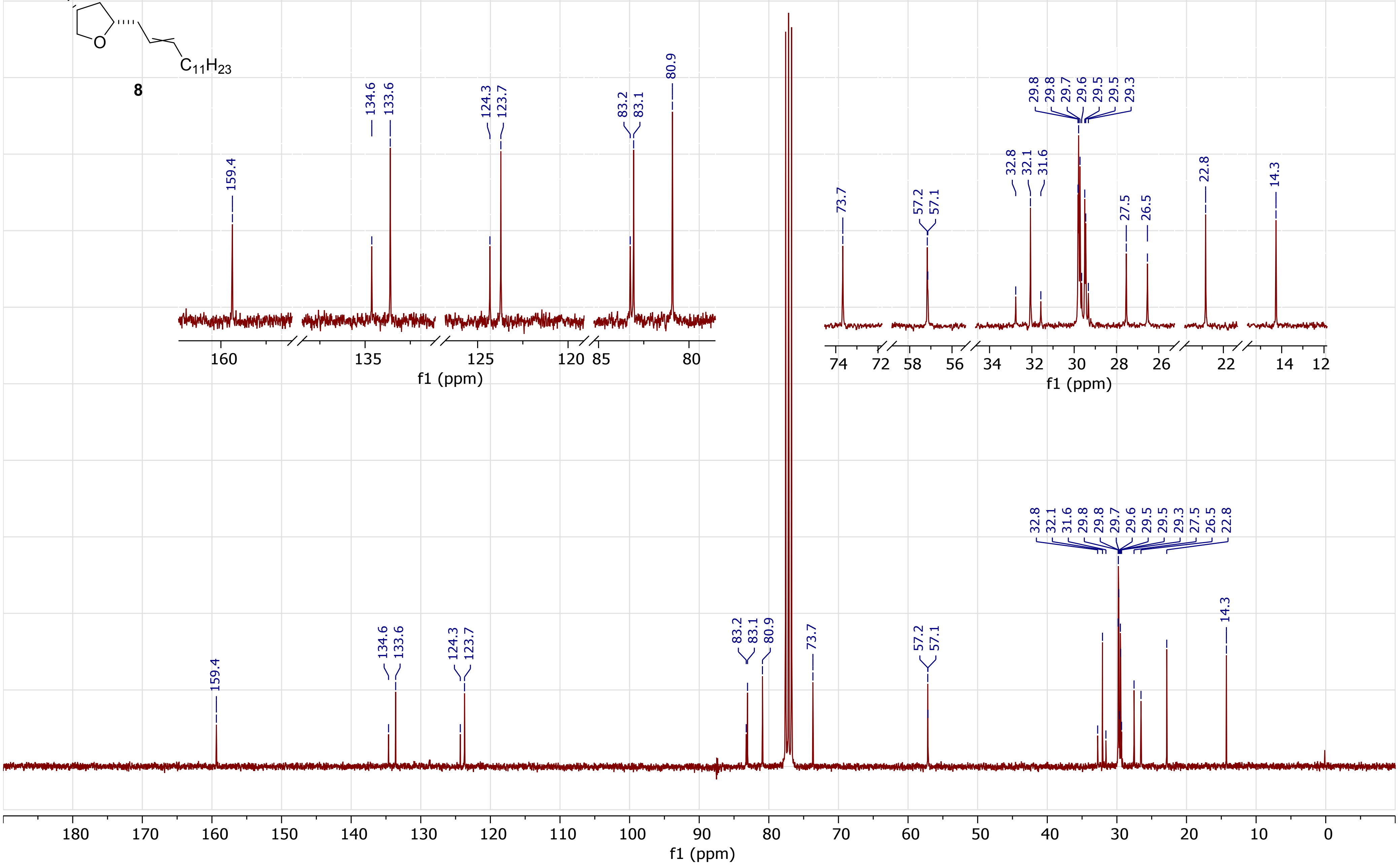

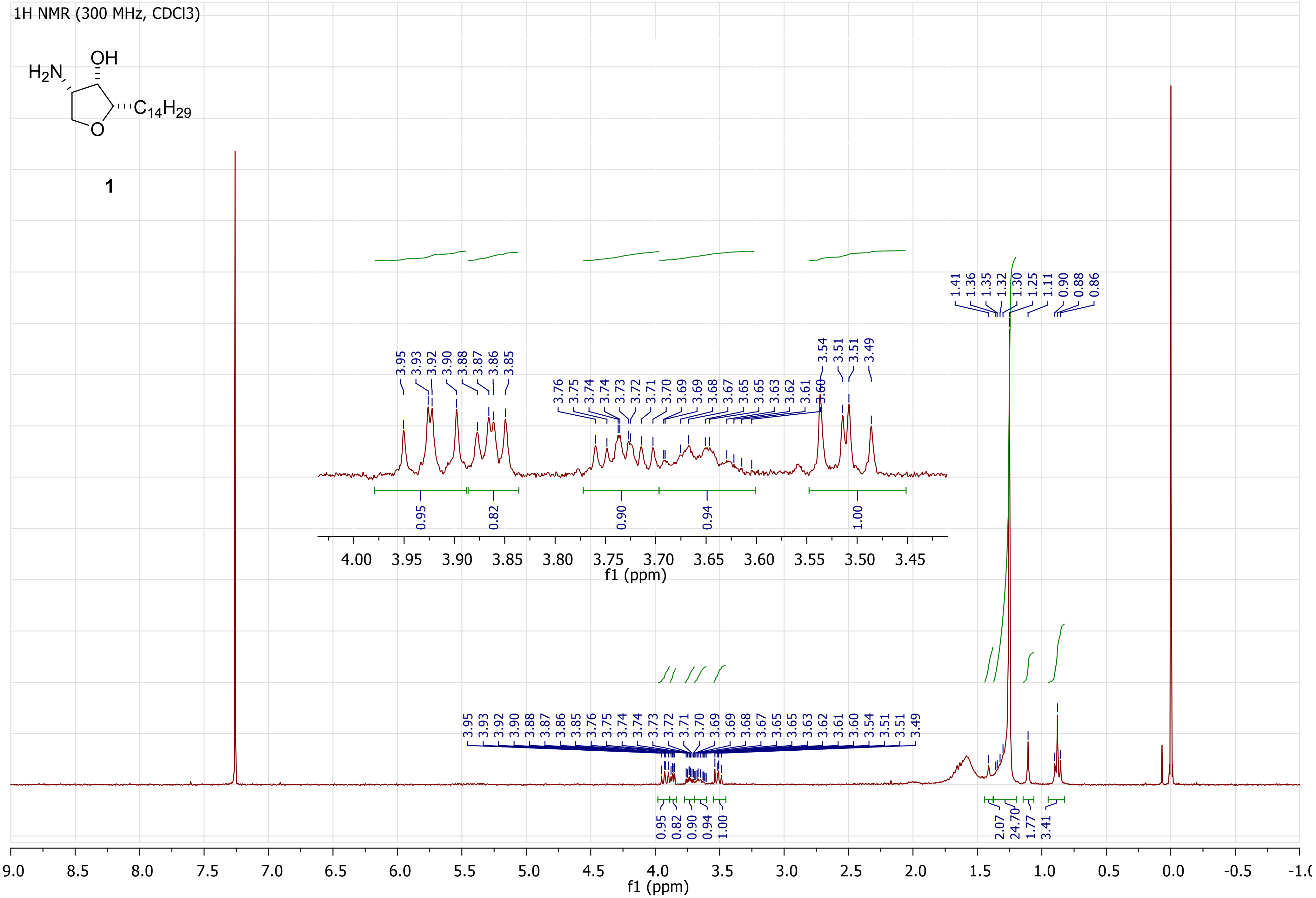
1H NMR (300 MHz, CDCl3)
H2N
OH
C14H29
O
1
f1 (ppm)
f1 (ppm)

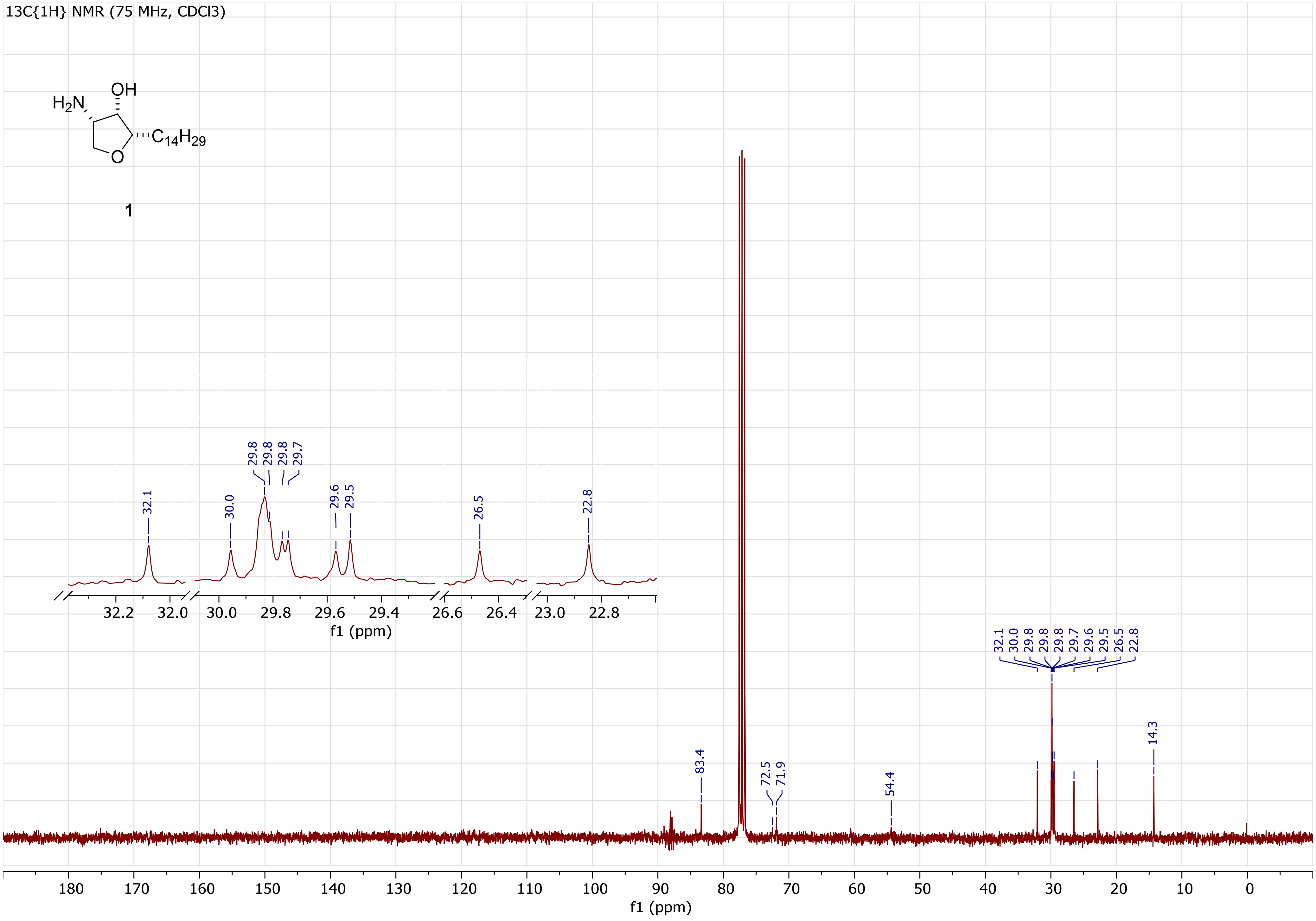
13C{1H} NMR (75 MHz, CDCl3)
1
32.1
30.0
29.8
29.8
29.8
29.7
29.6
29.5
26.5
22.8
14.3
83.4
72.5
71.9
54.4
f1 (ppm)
